# Leveraging the lipoprotein trafficking pathway for the development of novel antimicrobials

**DOI:** 10.1039/d6cb00009f

**Published:** 2026-04-14

**Authors:** Haley B. Gartrell, Taryn Trigler, Marcin Grabowicz, William M. Wuest

**Affiliations:** a Department of Chemistry, Emory University 1515 Dickey Drive Atlanta GA USA wwuest@emory.edu; b Department of Microbiology & Immunology, Emory University School of Medicine Atlanta GA USA marcin.grabowicz@emory.edu; c Emory Antibiotic Resistance Center, Emory University School of Medicine Atlanta GA USA

## Abstract

Antimicrobial resistance continues to limit the number of effective antimicrobials to treat bacterial infections. The development of antimicrobials with unique mechanisms of action is crucial to overcoming this threat. By employing various methods of hit identification, such as artificial intelligence, whole-cell screening, and drug repurposing, small-molecules that display activity against bacterial pathogens can be identified. Herein, four molecules (abaucin, enterololin, lolamicin, and fendiline) that were found to have high potency against either *Escherichia coli* or *Acinetobacter baumannii* utilizing these drug discovery strategies are described. They were all found to target the lipoprotein trafficking pathway (Lol). Within Gram-negative species, lipoproteins are essential for cell viability, making the Lol pathway an interesting target to exploit for the discovery of antimicrobials. This review highlights the importance of lipoproteins as antibacterial targets and details four examples of the development of small-molecule inhibitors of Lol.

## Abstract 2

The lipoprotein trafficking pathway and the known small-molecule inhibitors. The molecules depicted are enterololin, lolamicin, abaucin, and fendiline which target LolF and LolE within *A. baumannii* and *E. coli*.

## Graphical abstract

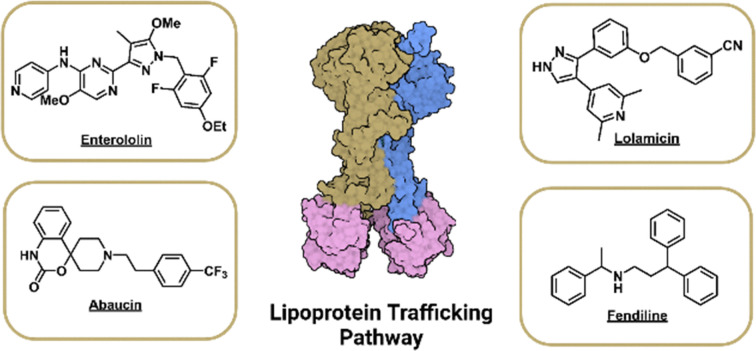

## 1. Introduction

With antimicrobial resistance on the rise, first-line therapeutics administered against deadly pathogens, such as Gram-negative bacteria *Acinetobacter baumannii* and *Escherichia coli*, are becoming ineffective. As a result, broad-spectrum second- and third-line treatments are being employed that commonly have harmful off-target effects. It is estimated that antimicrobial resistance will be the cause of over 10 million deaths per year by 2050 and on average cost the US over 55 billion per year.^1^

Antibiotic development has been severely limited over the past years due to low economic benefit. This has led to drug development focusing on manufacturing analog derivatives of existing antibiotic classes, rather than exploring novel mechanisms and molecular scaffolds. With diversity lacking amongst antibiotic candidates, resistance mechanisms can quickly adapt and render these derivatives ineffective. Gram-negative bacteria are amongst the most challenging to treat due to their cellular structure consisting of an additional membrane unlike Gram-positive bacteria leading to decreased drug uptake into the cell.^5^ Several common resistance mechanisms within Gram-negative bacteria are generally employed such as antibiotic target mutations, enzyme catalyzed modifications to the drug, and increased efflux pumps. The need for novel antimicrobial therapeutics with unique mechanisms of action (MOA) has become increasingly crucial to curb this global public health crisis.

**Fig. 1:**
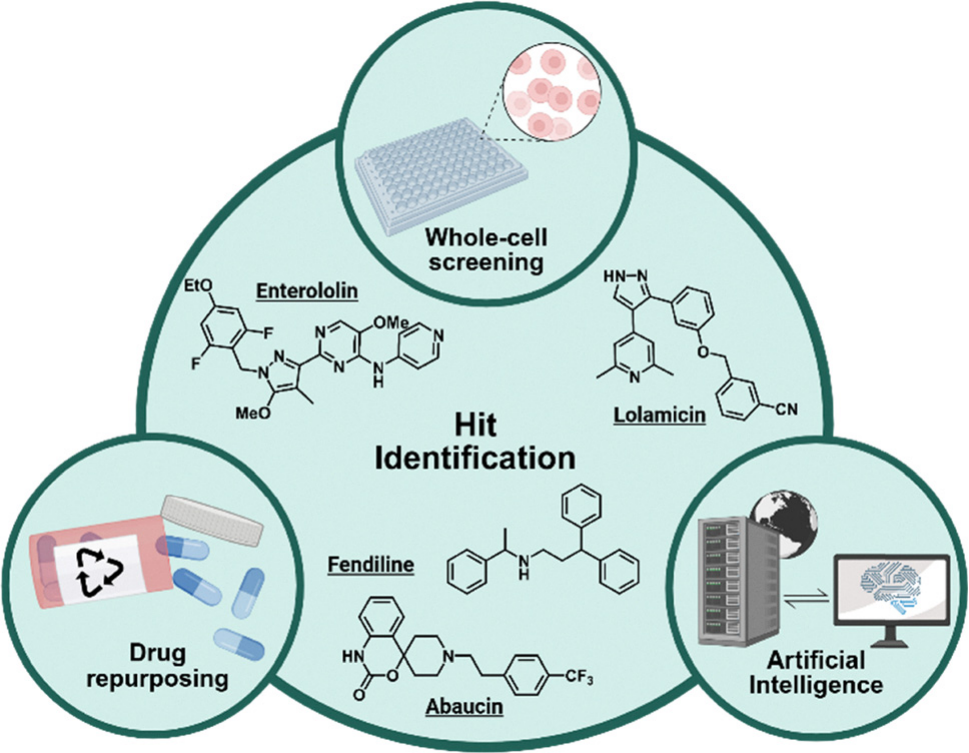
Overview of hit identification methods utilized to develop small-molecule antimicrobials. Created in BioRender. Lab, W. (2026) https://BioRender.com/f9gw8fl.

The challenge of discovering small-molecule antimicrobials has been approached using multiple avenues, most varying in their hit identification method. Main methods of hit identification include whole cell screening processes, drug repurposing screens, and the use of artificial intelligence or machine learning (Fig. 1). Using whole-cell assays is a popular method of identification due to the environments resembling the *in vivo* process closely.^2^ This process is generally carried out using live bacterial cells that are dosed with drugs from various small molecule libraries.^3^ While whole-cell screens are still widely performed, the utilization of drug repurposing techniques has been on the rise. Throughout drug repurposing techniques, FDA-approved drug libraries, which previously had no reported antimicrobial activity, are screened for hits that show potent activity against varying bacterial pathogens. This method of identification accelerates the drug development process by bypassing extensive safety and pharmacokinetic trials.^4^ Unlike both whole-cell and drug repurposing techniques, artificial intelligence and machine learning can leverage databases to sort through previously identified inhibitors of bacterial pathogens to note similarities and ensure novelty of the newly identified hit. In addition, it can help determine a compound's MOA by predicting properties such as binding affinity within the target. Each of these methods are valuable processes that can help with the discovery of antimicrobials that evade resistance pathways.

To circumvent the issue of permeability and lack of diversity amongst antibiotic targets, the exploration of new pathways has been at the forefront. Multiple advancements have recently been made toward the development of small-molecule inhibitors that target the Lol (localization of lipoproteins) pathway, located in the periplasm. Periplasmic targets are appealing in antibiotic drug development due to eliminating the need to pass through the inner membrane (IM). The Lol pathway is comprised of a 5-component protein system that transports mature lipoproteins (acylated proteins) from the IM to the outer membrane (OM).^3^ This pathway has been proved essential for bacterial cell viability and can be exploited to punish a variety of Gram-negative pathogens. The inner workings of the Lol pathway in differing Gram-negative species are continuously being explored to inform and direct drug campaigns.

In the last decade, several inhibitors of the Lol pathway have been discovered that show MOAs that evade resistance pathways, while also exhibiting potent antimicrobial activity within *E. coli* and *A. baumannii*. In the content of this review, we will outline the function of lipoproteins within Gram-negative bacterial cells, while also outlining the mechanics of the lipoprotein trafficking pathway. From there, the identification of several known inhibitors of Lol will be described and their drug discovery process. The Lol pathway continues to be an interesting target for antimicrobial development and future studies within the field could continue to provide solutions to the ever-growing antimicrobial resistance crisis.

## 2. Lipoproteins – Gram-negatives and Gram-positives

A subset of proteins that are secreted from the cytosol by bacteria are routed to an acylation pathway for their maturation.^6^ Collectively, these proteins are termed “lipoproteins” and can be identified by a characteristic “lipobox” signal sequence.^7^ An invariant cysteine in the lipobox will become the first (+1) amino acid of the mature lipoprotein and is the target of all acylation. Both Gram-positive and Gram-negative bacteria produce lipoproteins, but variations in structure exist.^6^ In *E. coli*, Lgt, LspA, and Lnt enzymes produce the mature, triacylated lipoprotein structure, and this lipidation allows anchoring of generally soluble, globular protein structures to the plane of a membrane.^8^ Several Gram-negative lipoproteins are essential for viability, making their maturation a viable therapeutic target.^9^ The natural product globomycin is a long-ago described LspA inhibitor, and more recent efforts have identified molecules targeting each maturation enzyme.^10–12^

*E. coli* falls in the Proteobacterial phylum, which is the best characterized of the Gram-negative bacteria. Given the universal conservation of the acylation enzymes, it seems likely that all Proteobacteria and perhaps even all Gram-negative phyla inherently produce the same triacylated lipoprotein structure as defined in *E. coli*.^9^ In stark contrast, some Gram-positive bacteria produce vastly more diverse and interesting lipoprotein structures, including peptidyl modifications of Cys^1+^, varying extents of acylation, and even acetylation.^6,13–16^ The need for some bacteria to generate such lipoprotein diversity is proposed to stem from their goal of evading mammalian innate immunity, which has evolved to be sensitively attuned for detecting fragments of the triacyl lipoproteins as hallmarks of active bacterial infection. Unlike in Gram-negatives, the lipoprotein maturation pathways in Gram-positive species are non-essential.

Among the diderm Gram-negative bacteria, mature lipoproteins can be localized in either the IM or the OM. At least in *E. coli*, the overwhelming majority of the ∼110 lipoproteins produced are OM localized, with only a few remaining in the IM.^9,17^ To reach the OM, lipoproteins must leave the favourable, hydrophobic IM lipid environment, be brought across a hostile aqueous periplasmic space, and then be inserted into the distant OM bilayer. Among Proteobacteria, almost all aspects of building the OM have come to rely on the actions of one or more OM lipoproteins, and other OM lipoproteins are important players in myriad pathways important for cell biology and, in the case of pathogens, the infection process.^9^ The broad essentiality of OM lipoproteins for the integrity of the bacterial cell and their importance for pathogenesis has made the Lol trafficking pathway that delivers them to the OM a particularly attractive target for novel therapeutics. Lol trafficking inhibitors are additionally intriguing because they would not only deprive the cell of essential components needed to build the essential OM organelle (the ultimate cause of bacterial cell death upon treatment), but they would also cause a back-up of OM-targeted lipoproteins in the IM, an event that for an increasing number of lipoproteins has been shown to result in lethal toxicity for the cell (an additional, proximate cause of death upon treatment).^18–20^

## 3. The localization of lipoproteins (Lol) system

The Lol system of *E. coli* was the first discovered and characterized, becoming the paradigm by which lipoprotein trafficking has been understood among diverse Gram-negative species.^21–23^ This system consists of LolABCDE proteins broadly responsible for recognizing OM-targeted lipoproteins, removing them from the IM, chaperoning them across the periplasm, and inserting them into the OM.^9^ In the IM, LolCD_2_E form an ATPase binding cassette (ABC) type transporter; LolC and LolE form a heterodimeric IM transporter complex with large periplasmic domains, and they pair with a dimer of cytosolic LolD ATPase. LolA is a periplasmic chaperone that ferries OM-targeted lipoproteins towards the OM. LolB is itself a lipoprotein in the OM that receives lipoprotein cargo from LolA and anchors its acyl chains into the OM bilayer.

**Fig. 2:**
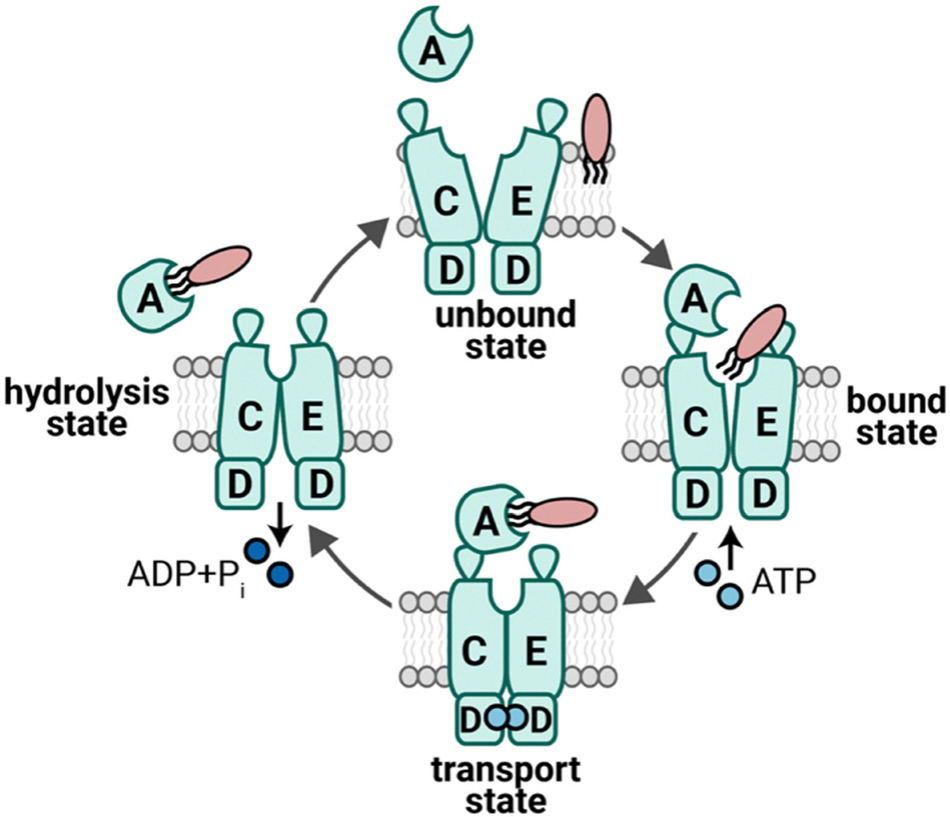
LolCD_2_E cycles to release lipoproteins to LolA, powered by ATP hydrolysis. LolCD_2_E lies in the IM of *E. coli* and releases lipoproteins from the IM for LolA-mediated transport to the OM by cycling through an unbound state, a bound state (in which ATP binds to LolD_2_), a transport state (in which the lipoprotein is handed off to LolA), and a hydrolysis state (in which ATP is hydrolyzed and the LolA–lipoprotein complex is released to the periplasm).

The IM LolCD_2_E transporter must accomplish several tasks to initiate OM lipoprotein trafficking (Fig. 2).^22,24^ It must discriminate which lipoproteins are to be trafficked to the OM from those that are to remain in the IM. It must provide power *via* ATP hydrolysis for extracting OM-targeted lipoproteins from the IM and offer a conduit for their movement out of the bilayer. Independently, it must recruit the LolA chaperone to which it will coordinate lipoprotein cargo handoff. Finally, the transporter must reset for a new round of work with the next lipoprotein. The dependence of multiple essential pathways on lipoproteins delivered by Lol and low abundance of LolCD_2_E transporter complexes in the cell are key features in the attractiveness of therapeutically targeting the Lol transporter with novel antibacterials.

As mentioned above, not all lipoproteins that *E. coli* produces localize to the OM. In fact, there are two prerequisites for OM localization through the Lol system. First, the lipoprotein must be triacylated.^16,25^ Second, the lipoprotein must bear the correct “sorting signal” amino acid sequence that discriminates IM-targeted *versus* OM-targeted lipoproteins.^24,26,27^ Curiously, starkly different sorting rules and mechanisms have been identified in diverse Proteobacterial species, and it is still unclear why no single mechanism appears to be universally favored.^9^

**Fig. 3:**
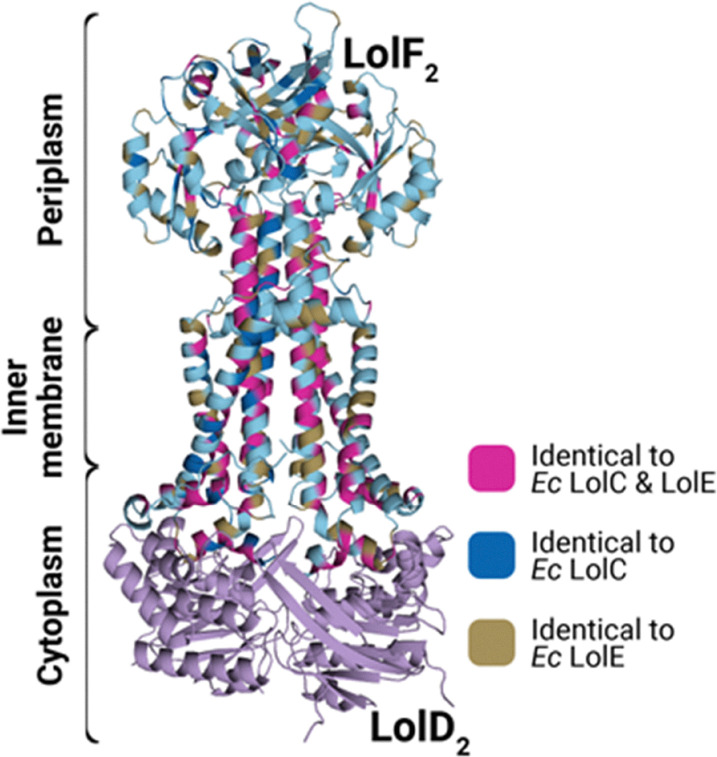
*A. baumannii* LolF features elements of *E. coli* LolC and LolE. *A. baumannii* LolF is chimeric. It is 37% identical to *E. coli* LolC and 38% identical to *E. coli* LolE. Identical regions are indicated by color on a LolF_2_D_2_ AlphaFold 2 structure prediction (pTM = 0.67).

In *E. coli*, the twin tasks of recruiting the LolA chaperone and preparing a lipoprotein for trafficking have been discretely delegated to LolC and LolE, respectively.^22,28^ Biochemical evidence supports a pathway for lipoproteins directly to a LolE binding site.^29^ Similar evidence, strengthened with crystal structure evidence, shows that a large periplasmic loop of LolC acts solely to recruit LolA.^22^ Meanwhile, LolD powers the cycling of LolCD_2_E from a conformation that permits lipoprotein entry into a binding pocket within the transporter to one which collapses the pocket, allowing the lipoprotein to be expelled to a recruited LolA. The extensive study of *E. coli* LolCD_2_E helped to form an elegant model. However, LolCD_2_E can be considered an outlier among Lol systems: many, and perhaps most, Gram-negative bacteria produce a different transporter with a different composition. Outside of Enterobacteriaceae, bacteria such as *A. baumannii* produce a LolF_2_D_2_ transporter where the IM transporter consists of a LolF homodimer paired to LolD_2_.^30,31^ The LolF protein sequence has hallmark features of both LolC and LolE and could be ancestral to the *E. coli* proteins (Fig. 3).^30^ The elegant division of labor between LolC and LolE is difficult to rationalize in LolF_2_ where each individual component has the same affinity for LolA and lipoproteins. How LolF_2_D_2_ functions in comparison to LolCD_2_E and how its functions are coordinated to achieve efficient lipoprotein trafficking remain open questions. A unique property of the LolF_2_D_2_ architecture is that these transporters seem to not absolutely require triacylation of the lipoprotein cargo; whether this is a laboratory quirk or reflects a capacity for specifically tuning lipoprotein maturation is still an open question.^30,32^

For *A. baumannii*, the Lol system and the LolF_2_D_2_ transporter are crucial for its renowned ability to broadly resist antibiotics. *A. baumannii* relies on both intrinsic OM impermeability and acquired mechanisms of resistance to key clinical antibiotics.^33^ As discussed, building the OM requires contributions from several essential OM lipoproteins that are delivered *via* Lol. *A. baumannii* also has several antibiotic efflux pumps that can expel broad classes of antibiotics, and these extensively rely on the oligomerization of several OM lipoproteins to form the efflux pore.^34^ Examples include OprM, AdeC, AdeH, and AdeK. Finally, some acquired resistance mechanisms employ OM lipoproteins in *A. baumannii*. The best studied of these is the carbapenamase OXA-23.^20^

## 4. Inhibitors of the lipoprotein trafficking pathway

Over the past 5 years, four main inhibitors of the Lol pathway have been discovered by research groups that focus on the development of antibiotics and understanding their MOA. Even though each of these inhibitors target the Lol pathway, they differ in specificity for different pathogens, as well as drug discovery processes. The two main Gram-negative bacteria that will be discussed are *E. coli* and *A. baumannii*. The inhibitors discovered are mainly narrow-spectrum antibiotics, a feature that has promise to salvage the gut microbiome. These include abaucin targeting *A. baumannii* and both lolamicin and enterololin punishing *E. coli* pathogens. However, more recently, a repurposed FDA-approved drug fendiline, has shown broad-spectrum activity against both Gram-negative species.

### 4.1. Abaucin

**Fig. 4:**
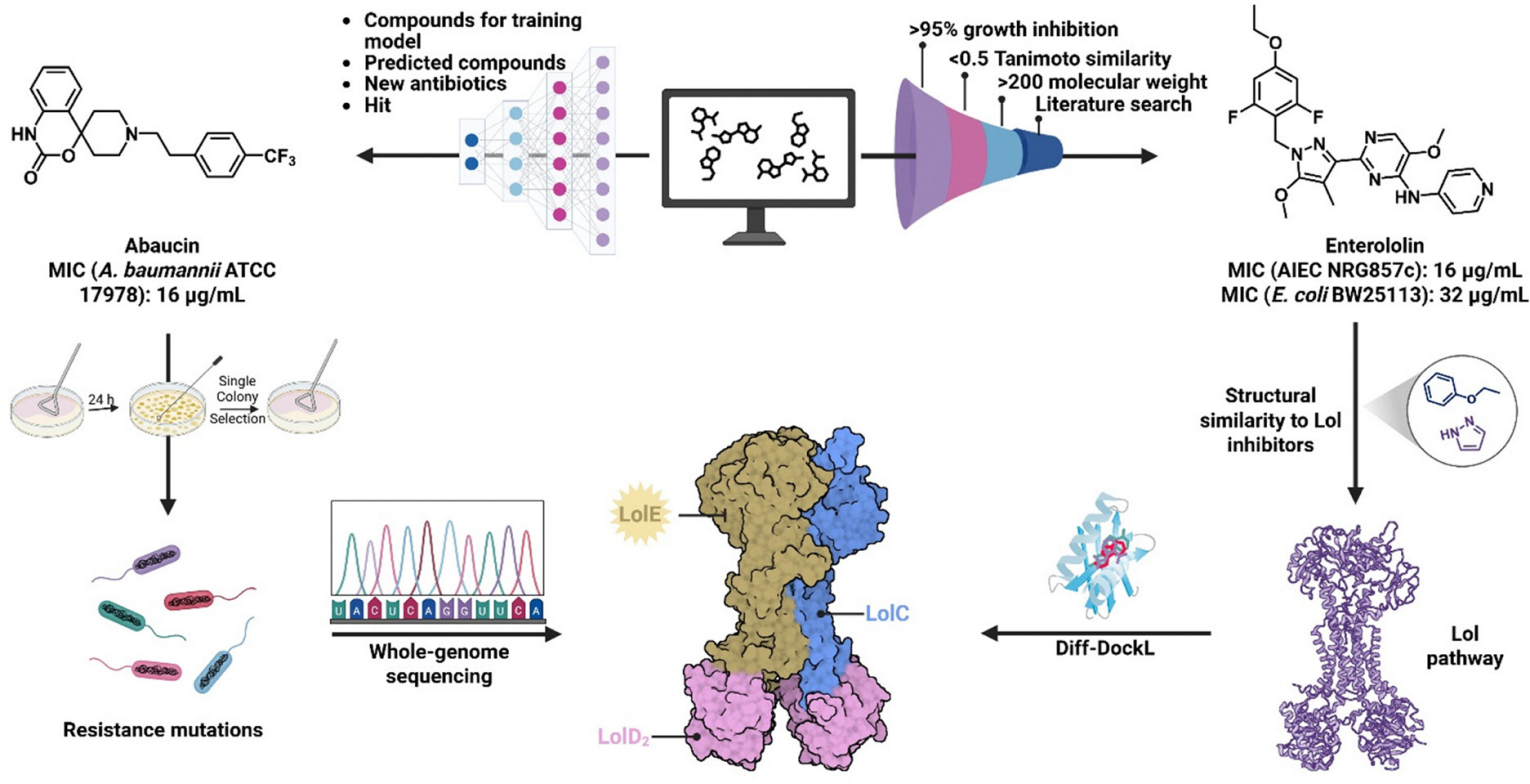
Overview of the discovery of abaucin and enterololin and identification of their target. Created in BioRender. Lab, W. (2026) https://BioRender.com/f9gw8fl.

A narrow-spectrum antibiotic of *A. baumannii*, abaucin, was discovered by the Stokes group in 2023 to have potent bioactivity, as well as target the Lol system.^35^ Throughout this publication, Stokes *et. al* exemplified the use of artificial intelligence (AI) and machine learning (ML) as a tool to accelerate the drug-discovery process. The start of their hit generation process began by screening a diverse library of small molecules against *A. baumannii* ATCC17978 for inhibition of growth to train their ML models. This allowed the ML model to classify molecules into two classes: active or inactive against *A. baumannii*. Once a broad characterization was made, further optimization of the model was achieved using a message-passing neural network (MPNN) architecture in tandem with a software toolkit, RDkit.^36^ With these implementations, the complexity of each small molecule was analyzed, as well as physiochemical and pharmacokinetic properties. With the model training complete, over 6,500 molecules were screened for potent antibactericidal activity against *A. baumannii* from a drug repurposing hub.^37^ Nine molecules were generated from the screen and subsequently diminished through the exclusion of those with similar structural features or reported bioactivity to existing molecules within literature, as well as compounds displaying non-specific membrane activity. Through the analysis of the priority molecules generated, the hit abaucin was found (Fig. 4).

Abaucin, a previously studied CCR2-selective chemokine receptor antagonist, was dosed against *A. baumannii* ATCC17978 to identify the compound's minimum inhibitory concentration (MIC) value. Against *A. baumannii*, abaucin has a MIC value of approximately 2 µg mL^−1^. Through further experimentation within different mediums, as well as the removal of abaucin from treated cultures, abaucin was determined to be acting as a bactericidal agent rather than bacteriostatic. A more extensive panel of Gram-negative species including *E. coli*, *P. aeruginosa*, and *S. aureus* were subjected to abaucin, and no potency was observed against these pathogens. Once abaucin displayed selectivity towards *A. baummannii* species, a wide range of *A. baumannii* clinical isolates were tested against. By performing a growth inhibition assay, abaucin was able to overcome or circumnavigate intrinsic or acquired resistance mechanisms present within each clinical isolate. As expected, when abaucin is used to treat carbapenem-resistant Enterobacteriaceae, *P. aeruginosa*, and *S. aureus* isolates no growth inhibition occurred. These further experiments helped prove once more that abaucin has selectivity towards *A. baumannii*, however, it was also established that the molecule is evading resistance pathways and has a unique MOA.

The mystery of abaucin's MOA started to be uncovered through a spontaneous mutation selection assay. Abaucin was revealed to have mutations located upstream of the gene encoding LolF (although referred to under an older annotation, LolE).^38^ The Lol pathway within *A. baumannii* is underexplored comparatively to the system within *E. coli*. However, it stands to reason that LolF_2_D_2_ of *A. baumannii* is functionally equivalent to *E. coli* LolCD_2_E, albeit varying in loosened requirement for triacylation of the lipoprotein. Further mechanistic insights were gained by Stokes *et al.* through a transcriptomics approach where abaucin was confirmed to disrupt the lipoprotein trafficking process and hence activate a two-component envelope stress response. Within this mechanism of action study, abaucin displayed a resistance frequency of 10^−8^–10^−7^*in vitro* which is comparable to antibiotic agents that have narrow-spectrum activity.

Due to previously observed narrow-spectrum activity within the clinical isolates and wild-type strain assays, abaucin was further tested against human commensals to determine if this is a potential solution for maintaining gut microbiota. Currently, patients prescribed antibiotics face multiple complications, generally associated with those that originate in gut infections. Abaucin was shown to avoid growth inhibition of a wide range of commensal species including skin and gut, but further *in vivo* studies need to be performed to confidently state the diversity of the microbiota is maintained. Additionally, abaucin's *in vivo* efficacy was tested by a wound infection model within mice. Within these studies, abaucin exhibits the ability to significantly reduce or suppress a wound infection within mice; however, this is only tested when the drug is directly applied to the wound. Further studies that explore how different drug delivery methods affect suppression need to be done. The discovery of abaucin shows how AI and ML guided discovery can help accelerate hit generation, as well as provide the field with a potent antimicrobial agent against *A. baumannii* that operated with a unique MOA targeting the Lol pathway.

### 4.2. Enterololin

After the discovery of abaucin as a potent narrow-spectrum bactericidal agent against *A. baumannii*, the Stokes group sought to discover more antibacterial small molecules that target the Lol pathway. Catacutan *et al.* focus was shifted from *A. baumannii* to the Enterobacteriaceae family, more specifically adherent-invasive *E. coli* (AIEC).^39^ AIEC is a Gram-negative bacterial pathogen that is commonly found within patients with inflammatory bowel disease, and it is becoming increasingly resistant to first-line treatments. Regardless of resistance build-up, treatment against AIEC is directly leading to the elimination of gut microbiota diversity. In contrast to the hit generation process the Stokes lab previously used, enterololin was discovered using a 4-step filtering process. Initially approximately 11 000 molecules were screened for growth inhibition of AIEC and then further narrowed down by molecular weight cutoffs, previously reported structural and physiochemical properties within literature. The sum of these eliminations resulted in the generation of enterololin.

Analogously to abaucin, enterololin is a repurposed small molecule, previously shown to be a Bub1 kinase inhibitor.^40^ When tested against AIEC NRG857c, a clinical isolate, enterololin exhibited an MIC value of 16 µg mL^−1^. Additionally, enterololin exhibited bactericidal activity against a lab strain of *E. coli*. Within Gram-negative pathogens, one of the main roadblocks to antibiotic development is permeation of the outer membrane. The MIC data generated against a clinical isolate and lab strain suggested that permeability was the crux of the issue. To test this hypothesis a hyper-permeable efflux-deficient *E. coli* strain was tested against. A 1024-fold decrease was observed when removing permeation and efflux out of the equation. Due to this issue, an antibiotic adjuvant, SPR741, was introduced and dosed in a combinatorial manner with enterololin against the clinical isolate strain of AIEC. SPR741 is a polymyxin B analogue that disrupts the outer membrane that is used *in vivo* and *in vitro*.^41–43^ Enterololin generated an MIC value of 0.03125 µg mL^−1^, identical to that within the genetically engineered strain of *E. coli*, suggesting this adjuvant as a helpful tool in navigating around permeation limitations. This goes to question whether abaucin's activity could be significantly reduced as well if dosed in combination with SPR741. Similarly to abaucin, Catacutan *et al.*, ran an extensive panel against other Gram-negative pathogens including *P. aeruginosa*, *A. baumannii*, *K. pneumoniae* and the Gram-positive *S. aureus*. These growth inhibition assays resulted mostly in low potency (128 µg mL^−1^) against each strain, except for *K. pneumoniae*. An MIC value of 16 µg mL^−1^ was reported for enterololin against *K. pneumoniae*. Unlike abaucin, which only possessed potent activity within *A. baumannii* strains, enterololin has preliminary specificity for the Enterobacteriaceae family which includes both *E. coli* and *K. pneumoniae*.

Further studies were conducted against antibiotic-resistant clinical isolates of Enterobactericeae in combination with SPR741 showing high potency, as well as displaying that resistance mechanism can be overcome. However, further assays against other Gram-negative species argued Catacutan *et al.* claim that enterololin's activity is narrow spectrum against Enterobactericeae species when in combination with an antibiotic adjuvant. Moderate activity against between 16–32 µg mL^−1^ was observed in the Yersiniaceae family, with testing of *S. marcescens* (LolCD_2_E producer). Hence, the narrow-spectrum activity is likely defined by the target homology rather than a broader phylogenetic clustering of species. Due to limited understanding of the Lol pathways of *A. baumaniii* and *S. marcescens* strains, it is currently difficult to rationalize why there is a difference in enterololin potency.

To test the hypothesis of enterololin targeting the Lol pathway, docking studies were performed to generate binding predictions within the LolCD_2_E complex.^44^ The predicted binding pose most probable was within LolE, the site of lipoprotein binding. LolF within *A. baumannii* similarly serves as the site of lipoprotein binding. Experimental validation of the predicted poses was conducted by evolving enterololin resistant mutants which were recovered in either LolC or LolE. To support their claim of narrow-spectrum activity, the frequency at which enterololin resistant mutations arose within an engineered strain of *E. coli* (10^−8^ to 10^−7^) were compared to other antibiotics with a single protein target. To their satisfaction, mutation frequency rates were found to be similar; however, the activity displayed within both *S. marcescens* and *A. baumannii* still leave the narrowness of enterololin's activity a question if it is a claim amongst species and not target homology.

Due to the focus of this drug identification effort being directed towards finding a remedy that treats AIEC infections within Crohn's disease patients, the potential of limiting dysbiosis was explored through *in vivo* and *in vitro* studies. Preliminary studies of enterololin's toxicity were conducted against human embryonic kidney cells at varying concentrations, which displayed no toxicity. From there several mice studies were employed that exhibited significant killing of bacteria within mice when dosed in combination with SPR741. Furthermore, AIEC-infected mice were treated with both enterololin alone and a combinatorial dose with SPR741 to generate data that suggests microbiome composition could be retained due to lack of killing of commensal bacteria. The Stokes group publications on abaucin and enterololin gave two strong examples of how the Lol pathway can be targeted with limited effect beyond key pathogenic bacteria.

### 4.3. Lolamicin

**Fig. 5:**
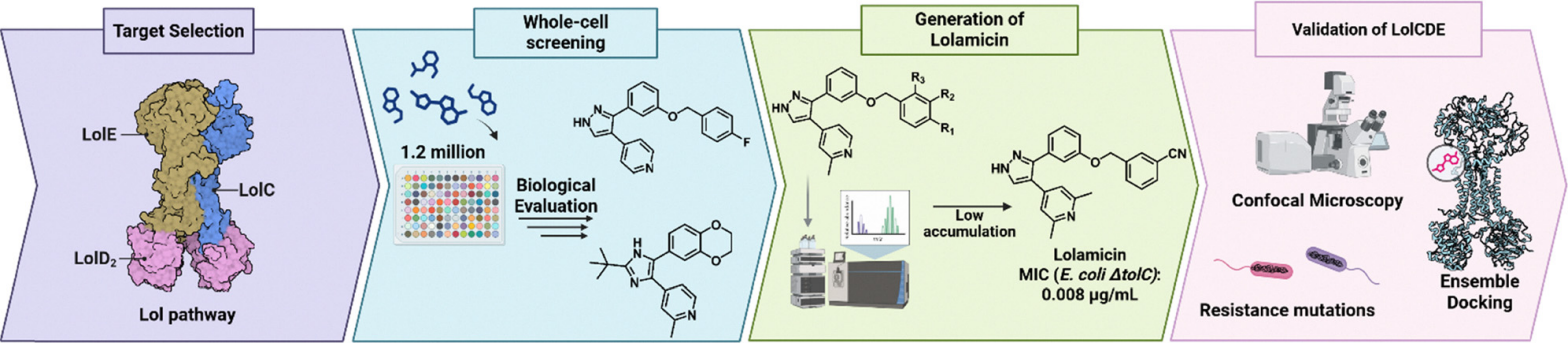
Overview of the discovery of lolamicin and identification of their target. Created in BioRender. Lab, W. (2026) https://BioRender.com/f9gw8fl.

Drug discovery campaigns, such as abaucin and enterololin, generally employ phenotypic screen processes to identify hit compounds. Phenotypic screens are typically conducted when no prior knowledge of the drug target is known.^45^ On the other hand, a genotypic screen is when the target is already identified, and a screen is conducted to discover compounds that directly inhibit it. Hergenrother *et al.* had prior knowledge about the Lol system being an essential system that is exclusive within Gram-negative bacteria. Additionally, Muñoz *et al.* was in search of a target where those commensal bacteria that produced the target did so with low sequence homology to the target in key Gram-negative pathogens, such as *E. coli.*^46^ The authors settled on LolCD_2_E as a target, for which prior whole cell screens already identified pyridinepyrazoles and pyridineimidazoles as inhibitors of the *E. coli* proteins (Fig. 5).^47^ These early compounds had early liabilities associated with them such as, lack of *in vivo* efficacy, poor solubility, low potency against wild-type strains, and high resistance frequencies. However, once tested against a hyper-permeable and efflux deficient strain of *E. coli* significant potency was observed suggesting that permeation and drug accumulation was the issue at hand. Unlike the other inhibitor campaigns, Muñoz *et al.* performed a small preliminary SAR study to attempt to improve intracellular accumulation through the addition of an amine.^48^ This addition resulted in increased whole cell accumulation, however drastically reduced target engagement with LolCD_2_E. To circumvent this issue, the two initial hit compounds were overlayed, and a hybrid scaffold was created. From the amine analogs data, they concluded that whole-cell accumulation was not required for activity since the LolCD_2_E was a periplasmic target which represents a less than 10% cell-volume.^49^ Accumulation assays were reperformed and compounds that were originally removed due to having no detectable accumulation were revealed and this process yielded lolamicin.

Lolamicin displayed initial activity against lab strains of *E. coli*, *K. pneumoniae*, and *E. cloacae*. This interesting display of activity supports the previous assumption made with enterololin that these pathogens have similar profiles against a Lol inhibitor due to producing very highly homologous LolCD_2_E complexes. In a wild-type strain of *E. coli* and *K. pneumonia*, lolamicin had a potency of 1 µg mL^−1^, with potency reducing slightly to 4 µg mL^−1^ within *E. cloacae*. However, potency drastically increased when tested against a hyper-permeable strain of *E. coli* and MIC values reached 0.008 µg mL^−1^. To assess lolamicin's full spectrum of activity, it was evaluated against a panel of multi-drug-resistant clinical isolates that consisted of *E. coli*, *E. cloacae*, and *K. pneumonia*. Lolamicin displayed potent activity ranging from 1–2 µg mL^−1^ against half of the multi-drug-resistant strains and the ability to kill all the strains with a minimum of 8 µg mL^−1^. Muñoz *et al.* did not explore the use of an antibiotic adjuvant in their paper, and it remains plausible that heightened potency of lolamicin could be achieved against WT strains if dosed with SPR741.

Even though a genotypic approach was utilized to generate lolamicin, the mode of action still needed to be verified. Resistance mutations were generated through exposing the bacterial strain to high concentrations of lolamicin. The mutations isolated were in LolC or LolE as expected and thereby confirming the Lol system as the target. From these resistance selection assays, the resistance frequency of lolamicin selection ranged from 10^−7^ to 10^−8^ amongst the three pathogens. Muñoz *et al.* further set out to determine whether lolamicin exhibited similar phenotypic signatures as other outer membrane inhibitors and they discovered that its signature was like that of globomycin and had no similarities to changes caused by β-lactams. The phenotypic similarities amongst lolamicin and globomycin further confirm Lol targeting since globomycin is a potent inhibitor of LspA which is needed for lipoprotein maturation, a prerequisite for Lol trafficking. To further characterize the LolCD_2_E binding site of lolamicin, ensemble docking was performed.^50^ The poses generated corresponded to binding sites in LolC and LolE and allowed for an in-depth analysis of nearby residues to lead future SAR studies. The binding site showed that hydrophobic interactions with surrounding non-polar or aromatic residues were favored and gave insight into why target engagement was lost when amines were added to previous scaffolds. Hergenrother's evaluation of lolamicin not only confirmed its target but allowed for the characterization of likely LolCD_2_E binding site to be further explored.

Muñoz *et al.* carried lolamicin throughout several *in vivo* studies to first analyze whether a reduction of bacterial burden within mice could be achieved. By dosing the mice with lolamicin on a strict regimen *via* intraperitoneal injections, lolamicin was found to be a potent antibiotic *in vivo*. Mice that were infected with a colistin-resistant *E. coli* isolate showed a significant reduction in bacterial burden when treated with lolamicin, as well as septic infection being fully resolved. Not only did they determine the efficacy of lolamicin through intraperitoneal delivery, but they also showed that oral delivery was an option. Similar experiments were conducted to determine how this administration relates to resistant *K. pneumoniae* strains and lolamicin remained effective. Mirroring results from enterololin and abaucin, lolamicin inhibition of the Lol pathway appeared to have very narrow-spectrum activity, which could be leveraged to create gut microbiome sparing therapies. Mice studies were once again utilized and the gut microbiome of healthy mice was analyzed following treatment with either a vehicle, a broad-spectrum antibiotic (amoxicillin), a Gram-positive antibiotic (clindamycin), or lolamicin. Large reductions in gut bacteria were seen in mice treated with clindamycin or amoxicillin; however, lolamicin treatment resulted in no significant changes throughout the treatment. Muñoz *et al.* went further and determined that *C. difficile* colonization—that is typically facilitated antibiotic therapies that disrupt the microbiome—is minimal during treatment with lolamicin. In all, Lol system inhibition appears to be ideal for treating Gram-negative pathogens, offering narrow-spectrum activity and allowing for preservation of gut microbiomes.

### 4.4. Fendiline

**Fig. 6:**
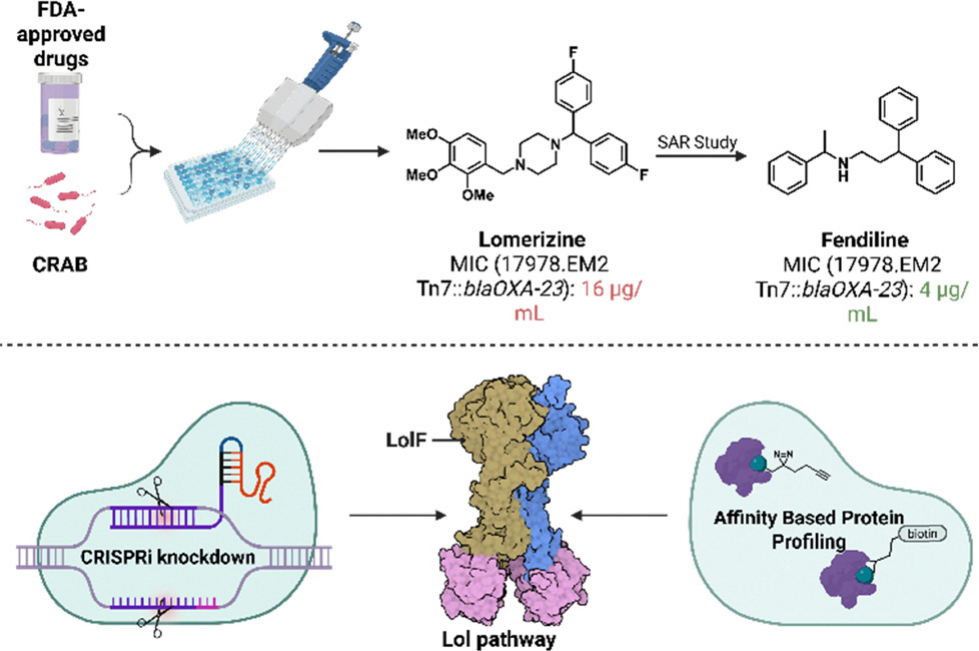
Overview of the Drug Repurposing screen performed against *A. baumannii* and the identification of the Lol pathway through CRISPRi knockdown and AfBPP. Created in BioRender. Lab, W. (2026) https://BioRender.com/f9gw8fl.

Drug repurposing techniques have been on the rise within the drug discovery realm, due to the increasing urgency for developing solutions for AMR. By utilizing drug repurposing techniques, safety trials and development of chemical profiles can be accelerated. The Stokes group paired drug repurposing with machine learning to identify both abaucin and enterololin. Likewise, in a recent collaborative paper, the Wuest group and colleagues performed a drug repurposing screening method to find small-molecule antimicrobial agents against carbapenem resistant *A. baumannii* (CRAB).^20^ Carbapenem resistance within Gram-negative pathogens is a rapidly rising issue that is mainly attributable to β-lactamases and their ability to irreversibly break open the β-lactam core scaffold.^51,52^ Notably, a common β-lactamase produced by *A. baumannii* is OXA-23. Previous studies performed by the Rather group showed that OXA-23 overexpression leads to additional fitness costs within the cell.^53,54^ This insight was implemented into a drug repurposing screen of over 3000 FDA approved drugs, targeting two strains of CRAB: wild-type and a hyper-permeable efflux-deficient strain. Using the latter strain, the screen sought to identify drugs with greater antibacterial effects when OXA-23 was induced to be overproduced compared to when it was transcriptionally repressed. An initial hit was lomerizine, an FDA-approved calcium channel blocker. Lomerizine displayed a potency of 128 µg mL^−1^ against *A. baumannii* not overexpressing OXA-23 compared to 16 µg mL^−1^ when OXA-23 was overexpressed, suggesting that OXA-23 overexpression does in fact lead to detrimental effects within the cell, and that these lead to lomerizine sensitivity. However, lomerizine had no activity against the wild-type strain suggesting that drug accumulation is a liability. Efforts towards finding a small-molecule with wild-type activity was continued by employing another screen searching for similar structural elements as lomerizine. The screen resulted in the hit fendiline, another previously known calcium channel blocker (Fig. 6).

Once fendiline was identified, an assessment of its activity against *A. baumannii* was conducted. Weak bactericidal activity of 128 µg mL^−1^ was still observed against a wild-type strain; however, against the hyper-permeable efflux-deficient strain, a dramatic increase of potency occurred and was exacerbated by overexpressing OXA-23, reaching a MIC value of 4 µg mL^−1^. A full panel against several pathogens (*A. baumannii*, *P. aeruginosa*, *K. pneumoniae*, *E. cloacae*, *S. aureus*, and *E. faecalis*) was conducted to determine the range of activity fendiline possesses. No wild-type activity was observed across the board, however further tests against these strains were conducted with the addition of differing adjuvants, such as a non-toxic Gram-negative permeabilizer (PMBN, polymyxin B Nonapeptide), colistin, and an inhibitor of a common efflux family, PaβN (phenylalanine-arginine β-naphthylamide). Notably the Gram-positive strains tested, *S. aureus* and *E. faecalis*, as well one Gram-negative pathogen, *P. aeruginosa*, showed no significant difference in potency with the addition of any adjuvant. This suggested that fendiline's target was not present within Gram-positive species, as well as certain Gram-negatives. Against *A. baumannii*, *K. pneumoniae*, and *E. cloacae* there is a 4-fold to 32-fold reduction in MIC observed which leads to the conclusion that drug accumulation is the issue surrounding this small-molecule (Table 1).

**Table 1:** Overview of biological activity of the Lol system inhibitors reported in µg mL^−1^. The abbreviation NT is used for categories not tested within the literature

Biological activity		Lol system inhibitors			
		Abaucin (µg mL^−1^)	Enterololin (µg mL^−1^)	Lolamicin (µg mL^−1^)	Fendiline (µg mL^−1^)
*E. coli*	Wild-type	>128	32	2	NT
	Engineered strain	NT	0.00313	0.008	16
	+ Potentiator	NT	0.00313	NT	NT

*A. baumannii*	Wild-type	2	>128	>128	>128
	Engineered strain	1	NT	NT	4
	+ Potentiator	NT	>32	NT	4

*K. pneumoniae*	Wild-type	NT	16	1–4	>256
	Engineered strain	NT	NT	NT	32
	+ Potentiator	NT	NT	NT	NT

Before conducting experiments to help determine the MOA of fendiline, a racemate of fendiline and each enantiomer was synthesized. Each synthesized molecule was re-tested to ensure the same antimicrobial profile was observed, however (*R*)-fendiline was 4-fold more potent than (*S*)-fendiline signifying there is possibly a protein-based MOA. To prove their hypothesis, affinity-based protein profiling (AfBPP) was performed by the appendage of a photoaffinity diazirine probe that enables photocrosslinking and a streptavidin pull-down. From this process, the Lol pathway was confirmed to be the target of fendiline, more specifically the LolF protein subunit. Further experiments, such as CRISPRi knockdown of LolF_2_D_2_ were performed to confirm the findings of the AfBPP process. The identification of the Lol pathway as the target of fendiline helped explain the antimicrobial profile previously observed, as well as open the door for activity comparison amongst other Lol inhibitors. Currently, it is not clear why fendiline is potent against both the LolCD_2_E producing Enterobacteriaceae family and LolF_2_D_2_ producing *A. baumannii*. MIC assays also showed that abaucin too showed a significant increase in potency with the induction of OXA-23 overexpression.

While Stokes and Hergenrother groups focused on finding a narrow-spectrum antimicrobial that could aid in preserving the microbiome, Wuest and collaborators aimed to explore the connection between carbapenem resistance and the Lol pathway. The hypothesis for why OXA-23 overexpression sensitizes cells to fendiline rested on the fact that OXA-23 is a lipoprotein and might therefore be transported the OM *via* the Lol system. Further studies, such as isolation of outer membrane vesicles, confirmed that OXA-23 was indeed a lipoprotein localized to the OM. Importantly, when OXA-23 is overexpressed and the Lol pathway is inhibited, toxicity within the cell rises due to OXA-23 mislocalization. Wuest and collaborators explored whether any of three other overexpressed β-lactamases (from different classes) could similarly sensitize *A. baumannii* to fendiline, but results showed the effect was specific for OXA-23. The insight that a mislocalized OM lipoprotein β-lactamase is toxic to bacteria could be implemented with other Lol system inhibitors. Overall, the Lol pathway is emerging as a particularly attractive target within Gram-negative species that can be leveraged to produce narrow-spectrum antimicrobials which evade common resistance mechanisms.

## 5. Future directions and outlook

Each of these drug discovery campaigns highlighted the novelty and usefulness of targeting the lipoprotein trafficking pathway, specifically the LolCD_2_E or LolF_2_D_2_ pathway. One common issue throughout the development of Gram-negative antibiotics is permeation through the double membrane cell envelope structure and the ability of the drug to avoid efflux and accumulate in the cell. Due to the Lol pathway being a periplasmic target, only the outer membrane needs to be crossed for inhibition. In addition, Stokes and Hergenrother groups elegantly demonstrated that by targeting this pathway, narrow-spectrum antibiotics could be generated that maintain the diversity and abundance of bacteria within the gut. The study of Wuest and collaborators additionally showed how carbapenemase resistance mechanism might enhance the lethal effects of Lol inhibition. While the development of Lol inhibitors is underway, researchers are still yearning to learn more about the lipoprotein trafficking systems within Gram-negative pathogens, such as *A. baumannii*. For example, currently there is no structure of the Lol complex of *A. baumannii*; with a more detailed view of the complex, better analysis and predictions could be made for antimicrobial development.

The future of Gram-negative antibiotics targeting the Lol pathway is bright and might be additionally helped with the use of combinatorial treatments with adjuvants such SPR741 that can break open the outer membrane to allow the drug to be shuttled into the periplasmic space. This may be a useful strategy that has the potential to be implemented into treatment through combinatorial dosing. Such adjuvants might also greatly help to expand the repertoire of Lol inhibitors that can be developed.

## Author contributions

Haley B. Gartrell: writing – original draft, review & editing. Taryn Trigler: writing – original draft, review & editing. Marcin Grabowicz: writing – review & editing, supervision. William M. Wuest: writing – review & editing, supervision.

## Conflicts of interest

There are no conflicts to declare.

## Data Availability

No primary research results, software or code has been included, and no new data were generated or analyzed as part of this review.
